# Automatic Detection Method of Dairy Cow Feeding Behaviour Based on YOLO Improved Model and Edge Computing

**DOI:** 10.3390/s22093271

**Published:** 2022-04-24

**Authors:** Zhenwei Yu, Yuehua Liu, Sufang Yu, Ruixue Wang, Zhanhua Song, Yinfa Yan, Fade Li, Zhonghua Wang, Fuyang Tian

**Affiliations:** 1 College of Mechanical and Electronic Engineering, Shandong Agricultural University, Tai’an 271018, China; zhenweiyu615@126.com (Z.Y.); szh6688@163.com (Z.S.); yanyinfa@sdau.edu.cn (Y.Y.); lifade@sdau.edu.cn (F.L.); 2 Shandong Provincial Key Laboratory of Horticultural Machineries and Equipment, Tai’an 271018, China; 2020120078@sdau.edu.cn; 3 Shandong Provincial Engineering Laboratory of Agricultural Equipment Intelligence, Tai’an 271018, China; 4 College of Life Sciences, Shangdong Agriculture University, Tai’an 271018, China; sffy521@163.com; 5 Chinese Academy of Agricultural Mechanization Sciences, Beijing 100083, China; ruixuewang@126.com; 6 College of Animal Science and Technology, Shangdong Agriculture University, Tai’an 271018, China; zhwang@sdau.edu.cn

**Keywords:** dairy cow, deep learning, DRN-YOLO, edge computing, feeding behaviour recognition

## Abstract

The feeding behaviour of cows is an essential sign of their health in dairy farming. For the impression of cow health status, precise and quick assessment of cow feeding behaviour is critical. This research presents a method for monitoring dairy cow feeding behaviour utilizing edge computing and deep learning algorithms based on the characteristics of dairy cow feeding behaviour. Images of cow feeding behaviour were captured and processed in real time using an edge computing device. A DenseResNet-You Only Look Once (DRN-YOLO) deep learning method was presented to address the difficulties of existing cow feeding behaviour detection algorithms’ low accuracy and sensitivity to the open farm environment. The deep learning and feature extraction enhancement of the model was improved by replacing the CSPDarknet backbone network with the self-designed DRNet backbone network based on the YOLOv4 algorithm using multiple feature scales and the Spatial Pyramid Pooling (SPP) structure to enrich the scale semantic feature interactions, finally achieving the recognition of cow feeding behaviour in the farm feeding environment. The experimental results showed that DRN-YOLO improved the accuracy, recall, and mAP by 1.70%, 1.82%, and 0.97%, respectively, compared to YOLOv4. The research results can effectively solve the problems of low recognition accuracy and insufficient feature extraction in the analysis of dairy cow feeding behaviour by traditional methods in complex breeding environments, and at the same time provide an important reference for the realization of intelligent animal husbandry and precision breeding.

## 1. Introduction

In modern smart farms, it is very important to monitor cows feeding behaviour and keep track of the movements of the cow’s head. Among them, the feeding behaviour can be used to predict whether dairy cows have diseases, and the changes in head movement during feeding can be used to evaluate feeding status and the quality of fodder. Studies have found that reduced feed intake and milk production are associated with health disorders [1]. Cows with mastitis have reduced feed and dry matter intake [2,3]. There is a striking difference between lame and non-lame cows in terms of feeding time, feeding frequency and feed intake [4], and based on this difference, the leg health of the cow can be judged. These studies have clearly shown that disease can have a significant impact on feeding behaviour. Monitoring the feeding behaviour of dairy cows is therefore an important tool to ensure intelligent husbandry and improve animal welfare.

There are two main research methods to detect cows feeding behaviour [5], one of which is to use wearable sensors to detect dairy cows’ behaviour and to collect physiological data, activity data and behavioural data through sensors installed in different parts of the cow, determining the type of cow behaviour based on processed data characteristics. The other is to use non-contact machine vision methods to detect cow behaviour [5], collect images and videos of cow behaviour through visual perception, and use foreground extraction, pattern recognition, artificial intelligence and other methods to process and analyse images and videos to achieve recognition of animal behaviour.

Traditional methods for identifying cow feeding behaviour have the following problems: (1) high resource consumption and high input cost; (2) monitoring quality is easily disturbed by ambient noise; (3) high rate of misjudgement of stress state in dairy cows. In recent years, with the continuous development and improvement of deep learning, deep learning methods have been widely applied to detecting and identifying cows’ behaviour [6]. The key to the studying of cow feeding behaviour lies in the accurate identification of different behaviours during cow feeding, and with the development of intelligent sensor technology, machine vision technology, and embedded edge computing technology, coupled with the advantages of deep learning artificial intelligence for data processing and image processing, by applying deep learning algorithms to the analysis of cow target detection, it can be judged whether deep learning can be applied to the detection and recognition.

In the farm environment, cows are only likely to feed when they are in the feeding area and their head is in contact with the feed; they are basically in the process of chewing the feed when their head is raised in the feeding area. Once they have consumed enough feed, they will leave the feeding area to lie down. Therefore, in the analysis of cow feeding behaviour, cows are divided into the following behaviours in the foraging area: (1) in feeding: this behaviour is where the cow’s head is in direct contact with the forage in the foraging area and there is a corresponding movement of the head to maximize access to the forage; (2) non-feeding/chewing: this behaviour is when the cow is in the feeding zone, lifts her head away from the feed area to chew, and the next action is either to continue feeding or to leave the feeding zone. Due to the need for accurate and rapid detection of dairy cow feeding behaviour, the two-stage detection network such as Faster Rcnn cannot achieve the requirement of rapid detection due to the cumbersome detection process.

Therefore, the main contributions of this paper are as follows: (1) based on the single-stage object detection network model YOLOv4, we seek to achieve enhanced feature information extraction by replacing the original YOLOv4 backbone network CSPDarknet with the self-designed backbone network DRNet, further adding a detection scale and using SPP structure to enrich the scale semantic feature interaction; we use CIOU Loss to achieve the screening of overlapping regression frames at detection, and finally a DRN-YOLO algorithm is proposed. (2) The DRN-YOLO algorithm is used to detect the feeding behaviour of dairy cows in real time, in order to achieve accurate and rapid monitoring of dairy cows’ feeding behaviour in the farm feeding environment and to lay the foundation for automated and welfare-oriented dairy farming.

## 2. Related Work

The most fundamental issue in techniques for monitoring and recording cow feeding behaviour is to accurately identify dairy cow feeding behaviour. It has been shown that real-time locating systems (RTLS) [7] and ultra-wideband (UWB) systems [8,9] can estimate the time cows spend in the foraging area to determine cow feeding behaviour. Although RTLS and UWB can determine how long cows spend in the foraging area, they cannot determine whether cows are feeding effectively. Other studies have monitored chewing movements by attaching pressure sensors to cows [10,11], and although the sensors have good monitoring performance, they are relatively cumbersome to use and inconvenient to put on cows. Shen Weizheng conducted a study on cow regurgitation using acceleration signals and edge computing techniques to achieve better recognition results [12]. Ear-mounted sensors based on inertial measurement units (IMUs) and radio frequency identification systems were used to position cows and monitor feeding behaviour [13], but only to determine the amplitude of cow movements, not whether cows were feeding effectively. However, the above studies are usually expensive and energy-intensive, and wearable sensors cannot accurately and effectively judge eating behaviours under long battery life. Tian et al. used a collar-type data acquisition system integrated with geomagnetic sensors and acceleration sensors to record data from cows in their daily activities, and classified cows’ feeding, regurgitation, running and other behaviours using the KNN-RF fusion algorithm with 99.34% classification accuracy, which can collect cows’ behavioural information quickly and continuously. The shortcoming is that the accuracy of this algorithm is limited by the accuracy of the sensors [14]. Campos et al. developed a surface electromyography-based approach to feeding behaviour recognition by combining multiple classifiers to achieve recognition of multiple metrics, including differentiation between grazing and regurgitation as well as recognition of different foods and combinations between foods, with an accuracy of over 70% for different combinations, through a multilayer perceptron neural network. Although this method recognises well, it requires shaving of the cow’s bite area, is not a non-invasive method of recognition and the device works with a disposable motor and has a rather limited working time [15].

The use of video cameras to record video and images of animals contains richer information, and by using image processing to achieve animal behaviour recognition and classification, animal welfare can be improved [6]. However, traditional machine vision extracts shallow features such as colour, texture, detection and recognition by artificial neural networks and threshold segmentation, with results dominated by manual analysis, which is not suitable for accurate identification of cow feeding behaviour. Deep learning has become one of the most effective methods in object detection [12]. Compared to traditional machine learning methods, deep learning avoids the need for manual annotation of features by autonomously learning the feature representation of an image. Deep learning object detection can currently be divided into two types of detection: single-stage detection and two-stage detection. YOLO [16] and Faster RCNN [17] are typical representatives of single-stage detection and two-stage detection, respectively. Single-stage detection combines the classification branch and the Region Proposal Network (RPN) branch into one network, using an end-to-end fully convolutional neural network to complete the input from the original image to the output of the prediction frame and the prediction frame category, improving computational efficiency. The two-stage detection uses separate RPN branches and classification branches to accurately locate the image object. Two-stage detection is more accurate than single-stage detection but much slower.

In recent years, deep learning has been widely used in animal husbandry. Achour et al. [5] used four CNN networks to achieve the detection of individual cow objects and feeding behaviour, but the models were relatively complex and required cooperation between models. Porto et al. [18] used the Violae-Jones algorithm and used multiple cameras to identify the feeding and standing behaviour of cows. Bezen et al. [19] designed a system for individual cow feeding measurements based on a CNN model and RGB-D cameras. Yang et al. [20] used full convolutional networks to extract sow head features and used the overlapping area between the head and the foraging area as spatial features to identify sow feeding behaviour. Lao et al. [21] developed an algorithm based on pre-processed depth image data for the study of sow feeding behaviour. Shelley et al. [22] devised a 3D image analysis method for measuring changes in the amount of food available in a feeder before and after a feeding period in dairy cows. The above research shows that the deep learning algorithm is feasible to study dairy cow feeding behaviour, but there is still no relevant research on distinguishing dairy cow feeding behaviour and tracking the feeding process. Therefore, it is necessary to identify and study dairy cow feeding behaviour.

## 3. Materials and Methods

### 3.1. Materials

#### 3.1.1. Edge Computing Device

**Figure 1:**
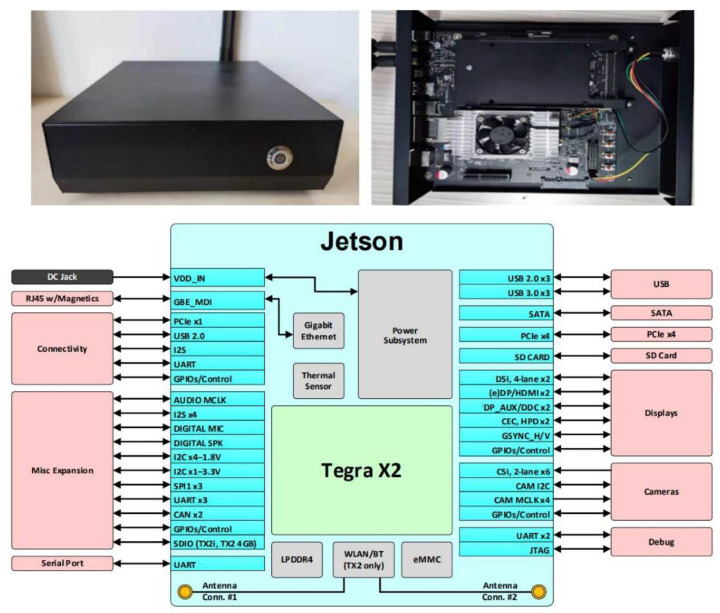
NVIDIA JetsonTX2 structure.

The NVIDIA JetsonTX2 is a single-module AI supercomputer based on the NVIDIA Pascal architecture, which is powerful enough to perform complex deep learning calculations, portable enough to carry around, and has a WIFI module to connect to the host system via a wireless network. In addition, the NVIDIA Jetsontx2 has the advantages of short response time, low energy consumption, easy movement, convenient installation, high calculation efficiency and lower cost. The NVIDIA JetsonTX2 is a fully enclosed metal case with a power-on button on the front and a WiFi module on the back, as shown in Table 1. The NVIDIA JetsonTX2 core board (under the cooling fan), the cooling fan, and the NVIDIA JetsonTX2 backplane are shown in Figure 1.

#### 3.1.2. Image Acquisition

**Figure 2:**
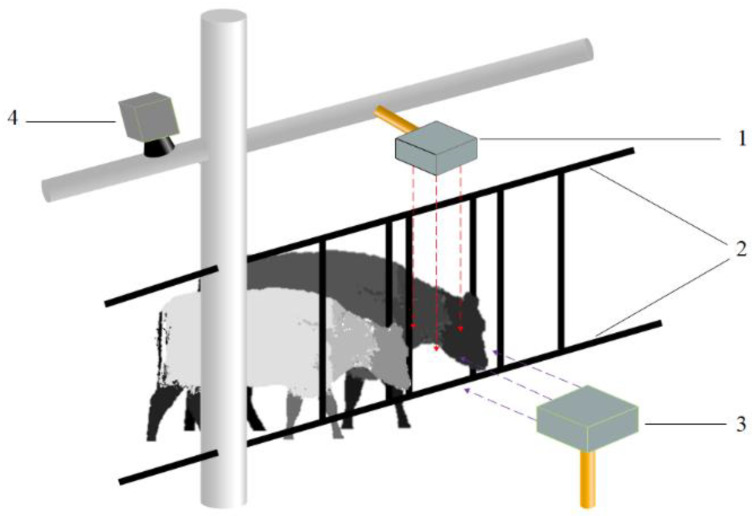
Equipment Installation Location. 1. ZED camera (top) 2. Cattle pen 3. ZED camera (front). 4. NVIDIA JetsonTX2.

The cow feeding behaviour dataset for this study was obtained from Taian Jinlan Dairy Farming Company in Nigou, Manzhuang Town, Daiyue District, Tai’an City, Shandong Province, China, which has a stock of over 2000 quality Holstein cows. This paper selected shed 3 as the test shed, which contained 17 lactating cows, aged between 1.5 and 2.5 years old, and in good condition. The test data were collected from 10 August 2021 to 5 September 2021, a total of 27 days, from 8:00 to 12:00 and 14:00 to 18:00 daily (with feed supplementation starting at 9:00–9:30 and 15:00–15:30 daily), to collect data on cow feeding behaviour. The ZED2 depth camera (STEREOLABS) was used for data collection. The ZED2 depth camera has a maximum viewing angle of 110°(H) × 70°(V) × 120°(D) and a maximum monocular resolution of 2208 × 1242 pixels, but the transmission frame rate is only 15 frames at this pixel. To ensure the stability and accuracy of the data collected on cow feeding behaviour, this paper uses a monocular resolution of 1280 × 720 pixels, at which a stable frame rate of 30 fps can be transmitted. As the top of the cattle pen is 1.35 m above the ground and the width of the feed belt is around 0.8 m, to avoid interfering with the normal feeding behaviour of the cows, the camera positions chosen in this paper are 1.75 m above the cow’s feeding area and 1.2 m in front of the cow’s feeding area at a height of 0.8 m, which is used to capture the feeding behaviour of the cows in different shooting directions, as shown in Figure 2.

#### 3.1.3. Dataset

**Figure 3:**
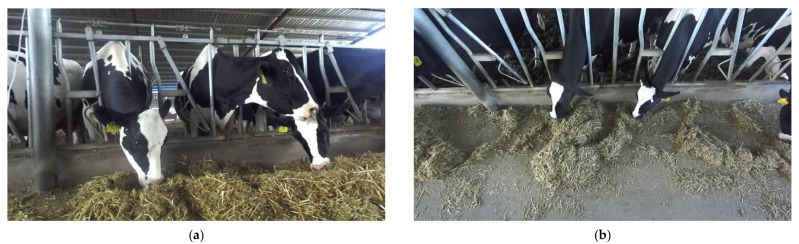
Dairy cow feeding behaviour. (**a**) Cow feeding behaviour from front; (**b**) Cow feeding behaviour from top.

In the farm environment, cows are only likely to feed when they are in the feeding area and their heads are in contact with the feed, as shown in Figure 3; when they raise their heads in the feeding area, they are chewing the feed and preparing to continue feeding; when they have consumed enough forage, they leave the feeding area. Therefore, the feeding behaviour of cows is divided into two main parts, feeding and chewing, where feeding can also be divided into two types of behaviour: feeding and pushing.

During the image acquisition process, a total of 20 sets of video data were collected, with one set containing four videos, one taken above and in front of the other from 8:00–12:00, and one taken above and in front of the other from 14:00–18:00. The ZED API was used to extract images from the videos frame by frame, removing duplicates, blurs, ghosts, and invalid images that did not contain the cows’ feeding behaviour, resulting in a total of 10,288 images in the dataset. The number of images taken from above was 5046, including 1263 images of one cow and 3783 images of multiple cows. In this paper, the open-source labelling tool LabelImg was used to manually label 10,288 original images of dairy cow feeding behaviours. In normal feeding, multiple dairy cows may compete for a piece of feed, which would cause their heads to overlap when feeding. Therefore, the following labelling rules were formulated: (1) Do not label the occluded and incomplete dairy cow heads; (2) label the adjacent dairy cow heads. LabelImg automatically generated the corresponding XML file every time a manual labelling of a dairy cow head was completed. The XML file recorded the coordinates of the upper-left corner and the lower-right corner of the labelling rectangle on the head of the dairy cow, that is, the feeding behaviour, the length and width of the labelling rectangle, and the labelling category information. Of these, the number of times each behaviour was represented in the dataset is shown in Table 2. 

### 3.2. Improved YOLO Model

#### 3.2.1. DRN-YOLO

**Figure 4:**
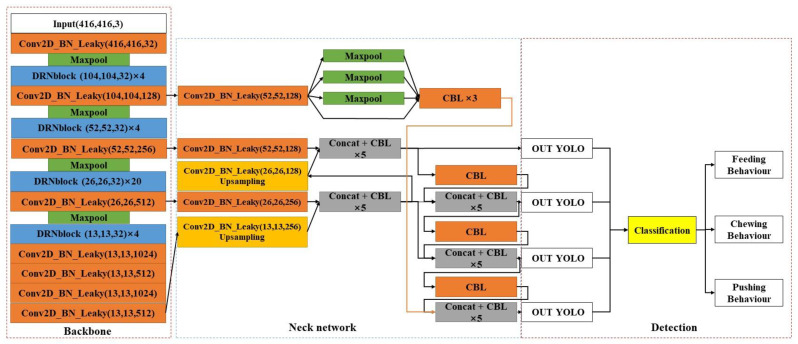
DRN-YOLO structure.

To achieve accurate and rapid detection of cow feeding behaviour in farm feeding environments, this study proposes a DRN-YOLO-based target detection framework for cow feeding behaviour in farm feeding environments, the specific structure of which is shown in Figure 4. The DRNet is used as the backbone network to extract features at four different scales of cow feeding behaviour, a feature scale linked to the backbone network is added to the neck network, and the SPP structure is used at the feature scale as a way to achieve the aggregation of local and global features of the feature pyramid and enrich the expression capability of the feature map. Finally, the feature map is sent to the detection module, and the detection module frames the output results in the feature map and selects whether to label the confidence, frame coordinates, and category information according to the program settings.

#### 3.2.2. DRNet

**Figure 5:**
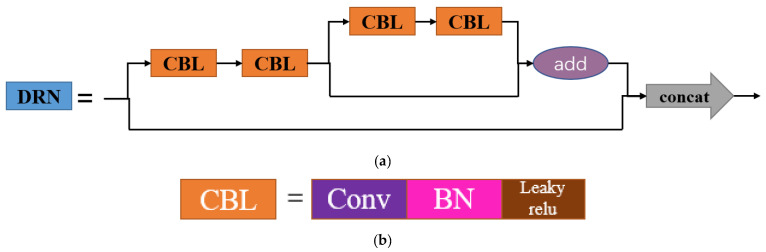
DRNet block structure. (**a**) DRN block; (**b**) CBL.

The DRN-YOLO algorithm model designed in this study uses the self-designed DRNet backbone network to replace the CSPDarknet backbone network of YOLOv4. The DRNet backbone network draws on the DenseNet [23] and ResNet [24] backbones and incorporates the Denseblock and Resblock modules. The DenseNet backbone network is composed of a CBL structure and a Denseblock module. With the assistance of the Denseblock module, the gradient disappearance phenomenon can be effectively reduced. While enhancing the feature map transfer, it also reduces the number of parameters to a certain extent, making the network detection faster; ResNet is composed of a CBL structure and a Resblock module. The DRNblock structure in the DRNet backbone is shown in Figure 5: it consists of two 1 × 1 CBL structures, two 3 × 3 CBL structures, a tensor summation layer, and two shortcut channels; when the feature information is passed from the upper part to the DRNblock, the information is passed from two paths to the next level, one path passes directly to the next level via a shortcut channel, the other path passes through a 1 × 1 CBL structure and a 3 × 3 structure first, after which the information is again passed down from the two paths, one path passes through the shortcut channel, one path passes through a 1 × 1 CBL structure and a 3 × 3 CBL structure again, the two paths are converged by an additional layer, and the information from the channel is tensor stitched and passed on to the next level. When the feature information passes through the DRNblock, on the one hand, the feature information is directly passed from the shortcut channel to the output location, protecting the integrity of the information; on the other hand, the model only needs to learn and reinforce the part of the input and output differences, and each layer directly connects the input and loss parts, simplifying the learning objectives and difficulties while also mitigating the gradient disappearance phenomenon. Therefore, compared with the original YOLOv4 backbone network, the model depth of the DRN-YOLO backbone network is deeper, the feature information extracted from the feature map is more comprehensive, and the learning ability is stronger.

#### 3.2.3. SPP Structure

**Figure 6:**
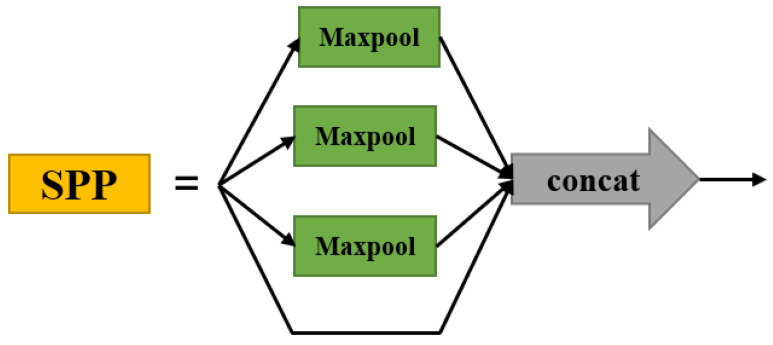
SPP structure.

The SPP [25] structure is shown in Figure 6. The input of SPP is a feature map of any size. After multiple pooling at different scales, the feature maps of each level after pooling are obtained and stacked, and finally a feature map with a fixed dimension output is obtained. As the SPP structure takes a feature map from different angles for feature extraction and then aggregation, it enhances the robustness and perceptual field of the algorithm, while increasing the accuracy of object detection. Because the SPP structure only requires the feature map to be input once, its detection speed is 24–102 times faster than the detection speed of the R-CNN structure [25]. The SPP structure enables feature map fusion of local and global features, enriching the expressiveness of the final feature map and thus improving the training AP.

As the feature scale of the new feature pyramid is connected to the front-end shallow network, the feature information of cow feeding behaviour is relatively small compared to the deeper network, and feature extraction may be missing. By adding the SPP structure between the new feature scale and the shallow network, we can fuse the local and global feature information of cow feeding behaviour, enrich the feature information of the shallow network and increase the perceptual field, improve the accuracy of cow feeding behaviour object detection, and increase the speed of detection. 

#### 3.2.4. CIOU Loss

As shown in Equation (1), the loss function in the training of the DRN-YOLO cow feeding behaviour object detection model includes loss of anchor frame position (*Loss*_CIOU_) and loss of confidence (*Loss*_conf_).

(1) $$L\mathrm{oss}=L{\mathrm{oss}}_{\mathrm{CIOU}}+L{\mathrm{oss}}_{\mathrm{conf}}$$

For the model prediction module, CIOU was used instead of GIOU (the ratio of the intersection and union of the predicted bounding box and the ground truth bounding box). Considering the influence of scale, distance, penalty term, and the overlap rate between anchor boxes on the loss function, the object frame regression became more stable. The formula is as follows:

(2) $$\mathrm{CIOU}=IOU-\frac{\rho^{2}(b,b^{gt})}{c^{2}}-\alpha v\;$$

(3) $$\alpha =\frac{v}{1-IOU+v}$$

(4) $$v=\frac{4}{\pi^{2}}{\left(\arctan\frac{w^{gt}}{h^{gt}}-\arctan\frac{w}{h}\right)}^{2}$$

(5) $$Loss_{\mathrm{CIOU}}=1-IOU+\frac{\rho^{2}(b,b^{gt})}{c^{2}}+\alpha v$$

where *c* represents the diagonal distance of the smallest enclosing region of the bounding box that contains both the predicted bounding box and the classification accuracy of the supervised learning training set; *w*, *h*, w*^gt^*, *h^gt^* respectively represent the width and height of the current detection frame and the width and height of the prediction frame of the next frame; *α* is a weight function; *v* represents the similarity of aspect ratio; $\rho^{2}(b,b^{gt})$ represents the Euclidean distance between the prediction centre point and the real frame; and 1-CIOU is used to obtain the corresponding loss.

The loss of confidence *Loss*_conf_ formula is as follows:

(6) $$Loss_{\mathrm{conf}}=\sum_{i=0}^{S^{2}}\sum_{j=0}^{B}K[-\log(p)+BCE(\hat{n},n)]$$

(7) $$BCE(\hat{n},n)=-\hat{n}\log(n)-(1-\hat{n})\log(1-n)$$

(8) $$K=1_{i,j}^{obj}$$

where *S* is the number of grids and *B* is the number of anchor boxes corresponding to a single grid. *K* is the weight, which is set as 1 if the object is present in the *j*th anchor box in the *i*th grid and 0 otherwise. $\hat{n},n$ denotes the model predicted and labelled actual category for the *j*th anchor box in the *i*th grid during training. *p* denotes the probability of the cow’s feeding behaviour occurring.

### 3.3. DRN-YOLO Working Process

**Figure 7:**
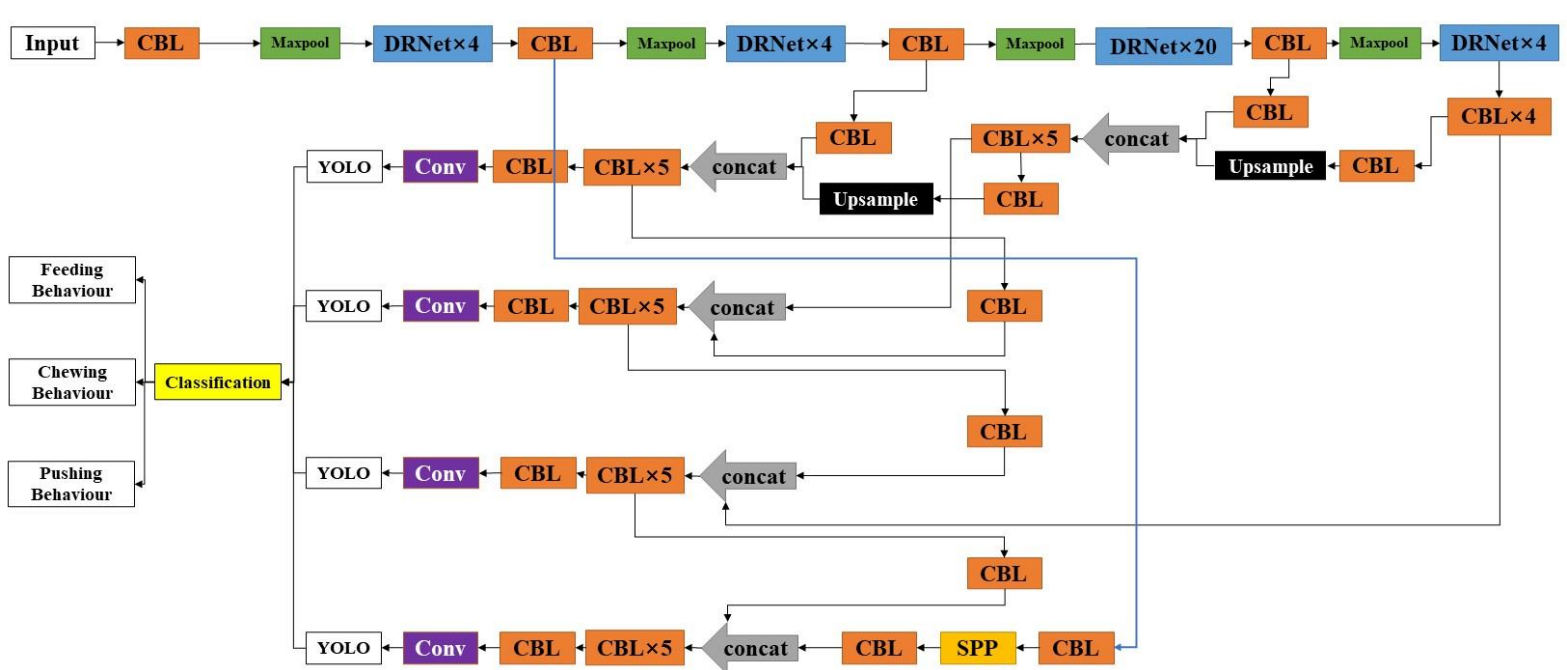
DRN-YOLOv4 model workflow.

The whole working process of DRN-YOLO is shown in Figure 7: a CBL structure, a maximum pooling layer, and different numbers of DRN modules form part of the backbone network; the input image goes through the backbone network part, consisting of 4, 4, 20, and 4 DRN modules in turn, and then enters the PAN path enhancement network [26], up-sampling after passing through the four-layer CBL structure and tensor stitching with the DRN (26, 26, 512) section and up-sampling and tensor splicing with the DRN (52, 52, 256) section after five layers of CBL structure to the first layer YOLO detection module. Then, the first-layer YOLO detection module detects the feature map and the DRN (26, 26, 512) part of the feature map for tensor splicing and then sends it to the second-layer YOLO detection module. The second layer YOLO detection module detects the feature map and the CBL (13, 13, 512) part of the tensor stitching to the third layer YOLO detection module, and finally the DRN (104, 104, 128) part is tensor stitched with the third layer. The YOLO detection module detects the feature map through SPP pyramid pooling to the fourth layer YOLO detection module. The final output target detection map is obtained.

## 4. Results

### 4.1. Evaluation Indicators

In order to accurately evaluate the performance of the model, this paper selects four common evaluation indicators in the target detection algorithms to test the performance of the model proposed: *precision*, *recall*, *mean average precision* (*mAP*), and F1-score to test the performance of the model proposed in this paper.

In terms of precision and recall, there are four states after predicting a test sample: true positive (*T_P_*), false positive (*F_P_*), true negative (*T_N_*), and false negative (*F_N_*), defined as follows:

(9) $$precision=\frac{T_{p}}{T_{P}+F_{P}}$$

(10) $$recall=\frac{T_{p}}{T_{P}+F_{N}}$$

Both *mAP* value and F1-score can be used to evaluate the performance of the object detection model. *mAP* and F1-score are defined as follows:

(11) $$AP=\int_{0}^{1}precision\cdot recall\;drecall$$

(12) $$mAP=\frac{1}{m}\sum_{m}AP$$

(13) $$F_{1}=2\times \frac{precision\cdot recall}{precision+recall}$$

In addition, the changing trend of the loss curve of the model can also be used to evaluate the pros and cons of the model. The faster the loss curve fitting speed, the better the fitting degree and the lower the final displayed loss value, which represents stronger performance of the adopted model.

### 4.2. Test Configurations

Among the test parameters, the size of the number of batch images (*Batch-size*) and the degree of subdivision (*Subdivision*) will affect how long training takes; the larger the value of the *Batch-size* and the smaller the value of the Subdivision, the shorter the time taken for training. We found that when the Batch-size exceeds 16 and the Subdivision is less than 8, the system terminal will show CUDAError: out of memory. Therefore, the value of the *Batch-size* is 16 and the value of the *Subdivisions* is 8. In YOLOv4, the width and height of the input image should be divisible by 13. The three commonly used image input sizes are 416 × 416, 520 × 520, and 608 × 608. In this study, the input image size of 416 × 416 is chosen to save computational costs.

**Figure 8:**
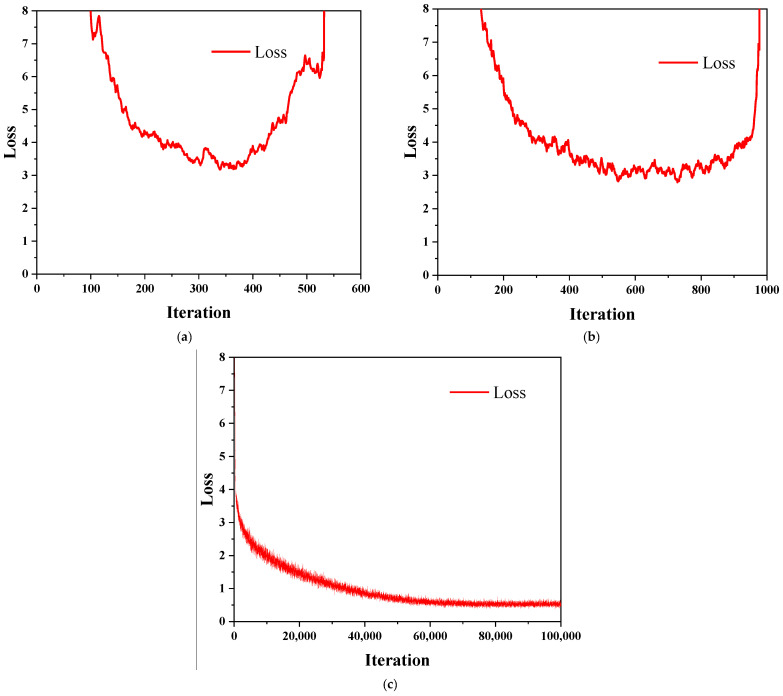
Impact of learning rate. (**a**) Learning rate 0.1; (**b**) Learning rate 0.01; (**c**) Learning rate 0.001.

Learning rate and weight decay rate are two hyperparameters in deep learning. The learning rate determines whether and when the loss function can converge to a local minimum. The learning rate must be appropriate. If the learning rate is too large, the loss function will fail to converge. Otherwise, the training time will increase significantly. When the value of the learning rate is 0.01, as shown in Figure 8b, the value of the loss function suddenly increases and fails to converge in about 620 iterations, and when the value of the learning rate is 0.001, as shown in Figure 8c, the value of the loss function converges well and does not show explosive growth, which meets the expected convergence requirement, so the initial value of the learning rate is set to 0.001; the value of the weight decay rate is equivalent to the L2 parametric regularization, and by adding a penalty term to the loss function, the aim is to reduce the overfitting problem by decaying the weights to a smaller value, which should be close to 0. The default value of 0.0005 meets the requirement, so the value of the decay rate of weights is set to 0.0005.

The maximum number of training iterations is directly related to the training time and the training effect, and under the condition that the initial value of the learning rate is 0.001 and the decay value of the weights is 0.0005, the influence of the number of iterations is tested by pre-training. The curve of the loss value was found to be under-fitted at 1000 iterations, with a value of the loss of 2.3167, and at 10,000 iterations the curve of the loss value started to converge but was still under-fitted, with a value of the loss of 1.2695. When the number of iterations reaches 40,000, although the curve still appears to be under-fitting, it is close to a perfect fitting state at this time, and the loss value is 0.5061; at 80,000 iterations, the curve basically reached a perfect fit, with a value of the loss of 0.3856. The value of the loss is 0.3745, which indicates that the model training has reached a perfect fit, so the maximum number of iterations is set to 100,000.

### 4.3. Testing Results

As the farm environment where the dataset was collected was non-open air, the effect of weather factors such as cloudy and foggy days on the test results was not considered in this paper, and the tests did not consider night-time cow feeding, so night-time detection did not appear in the identification results. DTCS labels indicate feeding behaviour, TTJJ labels indicate chewing behaviour, PTGC labels indicate pushing behaviour, and Land for open space is detected in the image.

**Figure 9:**
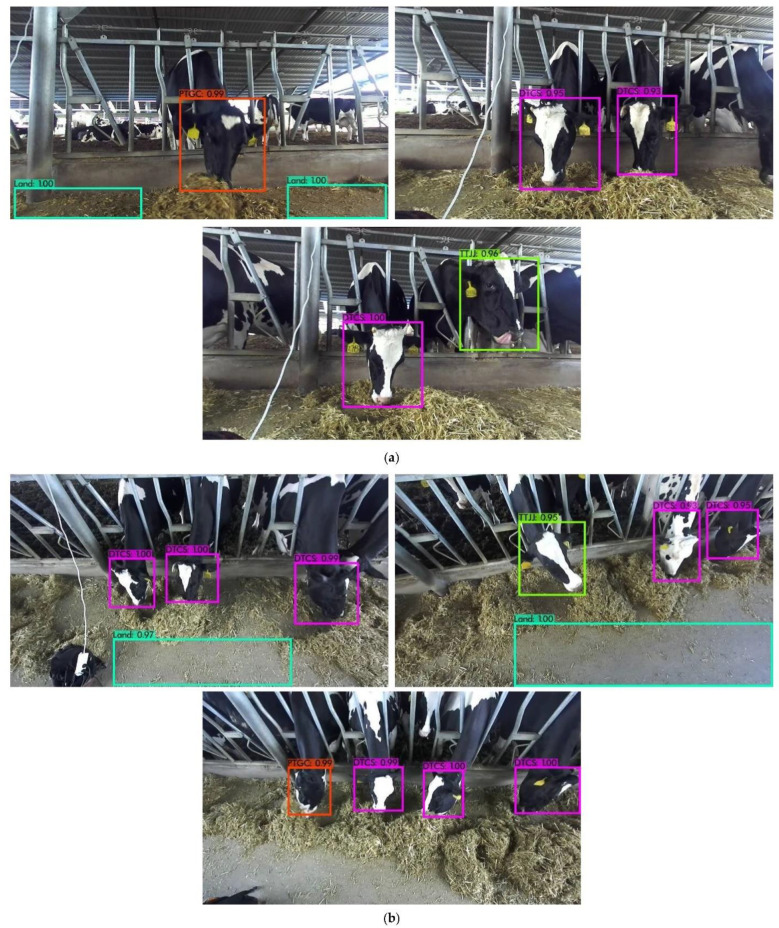
The results of DRN-YOLO identification. (**a**) Recognition results collected from the front; (**a**) Recognition results collected from the front; (**b**) Recognition results taken from top.

Figure 9a shows the identification results of the cow feeding behaviour test set taken from the front, and Figure 9b is the recognition result of the dairy cow feeding behaviour test set photographed from above. As can be seen from the figures, the DRN-YOLO model can accurately identify cow feeding behaviour with a confidence level above 0.96.

**Figure 10:**
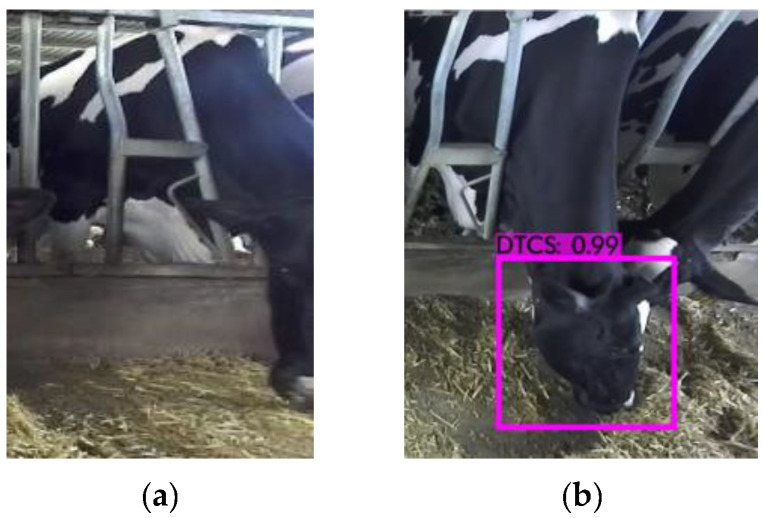
DRN-YOLO missing detection. (**a**) Incomplete shot; (**b**) Obscured from each other.

As can be seen in Figure 9, the DRN-YOLO model detected well, but cows are herd animals and will inevitably obscure each other. For example, Figure 10a shows a situation where only half of the cow’s head is captured during the collection process, and Figure 10b shows cows obscuring each other during feeding behaviour in the foraging area. It can be seen that both occlusions remove most of the features from the cow’s head, leaving only a small portion of the cow’s head, resulting in the model not recognising the cow’s feeding behaviour in the occluded portion during the detection process.

### 4.4. Ablation Test

Deep learning–based detection methods have been applied to many object detection tasks by scientists. This study is based on the YOLOv4 model, combined with different modules to verify the performance of the model and train the data sets on the feeding behaviour of dairy cows, captured and collected from the front and the top. The results are shown in Table 3 and Table 4. The tests show that the improved method has a degree of detection that is greater than the YOLOv4 model. Next, this study analyses the experimental data to reflect the enhancement of the model detection performance by the different improved methods in the DRN-YOLO model.

## 5. Discussion

### 5.1. Model Feature Map Analysis

In the detection process, the data of each convolutional layer are superimposed by multiple feature maps’ information. The feature map is the most representative part of the image features extracted by the convolutional layer. The feature information extracted from the feature map directly determines the merit of the model. This study uses the model to extract feature maps from the images of the cow feeding behaviour dataset captured and collected from the front and above, and further analyses the quality of the model based on the feature information.

**Figure 11:**
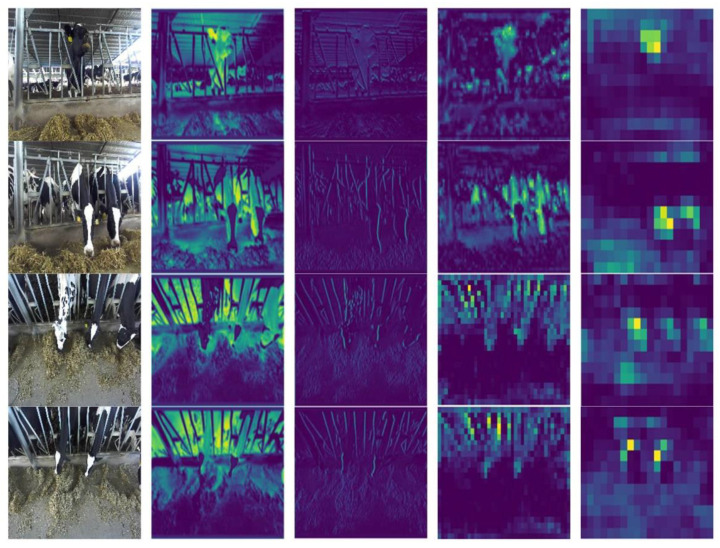
The features of dairy cow feeding behaviour.

Due to the different sizes of the feature maps, some of the feature maps cannot be identified manually, so several representative feature maps were selected. The DRN-YOLO feature maps of different layers are shown in Figure 11. It can be seen that as the network continues to deepen, it places more and more emphasis on the semantic representation of the object, and the feature maps become more abstract. The computer has the advantage of processing high-dimensional deep semantic feature information to achieve accurate recognition. 

Based on the DRN-YOLO model, the perceptual field of the feature map was increased to enrich the feature information. In Figure 11, the brighter the colour of the area, the more concentrated the features are in the area during the detection process. After extracting the feature information through the model, the feature map in front of the detection module only highlighted the feature information of the cow’s head during feeding, effectively verifying the effectiveness of the algorithm. Once again, the accuracy and effectiveness of this study’s algorithm for extracting features from cow feeding behaviour were demonstrated.

### 5.2. Comparative Performance Analysis

#### 5.2.1. Performance Comparison of Characteristic Scales

The detection of cow feeding behaviour requires adequate feature extraction of the behaviour currently performed by the cow and thus accurate judgment, while the YOLOv4 model has insufficient feature extraction ability, which is most intuitively reflected in the low mAP value of the model. To enhance the feature extraction of cow feeding behaviour, we used an enhancement to the feature pyramid network [27], i.e., adding a feature scale that allows for a closer connection between the deeper network in the backbone network and the neck connection network. It can be seen from Table 3 and Table 4 that when the new feature scale is added, the mAP value of the training data set of cow feeding behaviour photographed in front increases from 95.13% to 95.86% when the training is completed, and the mAP value of the cow feeding behaviour training data set photographed above increases from 95.01% to 95.53% after the training was completed, and the *accuracy* and *recall* were improved to varying degrees. By increasing the feature scale to extract features of the current feeding behaviour of cows, the detection failure rate of cow feeding behaviour was reduced and the model detection performance was improved.

#### 5.2.2. Performance Comparison of SPP Pooling Structures

It can be seen that increasing the *mAP* value to a four-feature scale increases the connectivity between the networks and enhances the ability to extract features from the behaviour of cows during feeding, but the feature perceptual field is not sufficiently extracted, making the feature extraction not comprehensive enough, so the SPP pooling structure was tested to solve the problem of insufficient feature extraction. The use of the SPP pooling structure allows the shallow network to be richer in feature information and to increase the perceptual field, thereby improving the model’s detection performance. With the addition of the SPP pooling structure, the *mAP* of the training dataset of cow feeding behaviour taken in front was 96.27%, 1.14% higher than the *mAP* of YOLOv4, and the *mAP* of the training dataset of cow feeding behaviour taken above was 95.97%, 0.96% higher than the *mAP* of YOLOv4.

#### 5.2.3. Performance Comparison of DRN Modules

The use of a four-feature scale and SPP pooling structure improved the model’s detection effect and made the detection of cow feeding behaviour more accurate, but adding structure makes the model bulkier. In order to reduce the complexity of the model and improve its depth, the CSPDarknet module in YOLOv4 is replaced with a DRN module. When the feature information passes through the DRN module, on the one hand, the feature information is passed directly from the shortcut channel to the output location to protect the integrity of the information. On the other hand, the model only needs to learn and reinforce the part of the input and output difference, which simplifies the learning objective and difficulty while also mitigating the gradient disappearance phenomenon and reducing the memory consumption used by the model. Using the DRN module alone, the *mAP* value for the training dataset of cow feeding behaviour taken in front was 96.16% when training was completed, 1.03% higher than the *mAP* value for YOLOv4, and the *mAP* value for the training dataset of cow feeding behaviour taken above was 95.69% when training was completed, 0.68% higher than the *mAP* value for YOLOv4.

### 5.3. DRN-YOLO vs. YOLOv4

**Figure 12:**
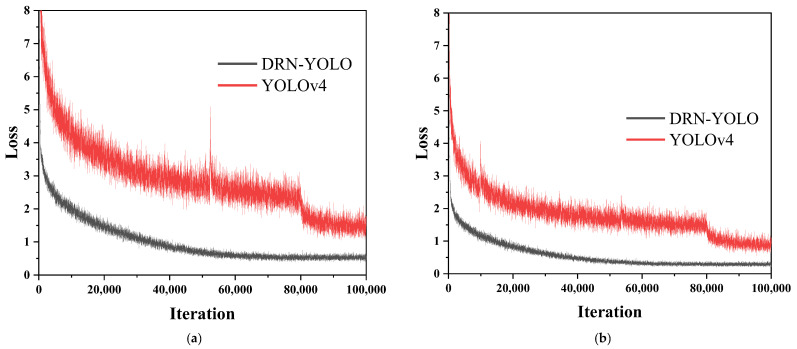
Training loss curves. (**a**) Training loss curve of the front; (**b**) Training loss curve of the top.

The above analysis shows that DRN-YOLO uses the DRN module and the SPP structure on an increased feature scale, and the model performance data is improved compared to the YOLOv4 model. The following is a comparison of the DRN-YOLO and YOLOv4 models in terms of the model value of loss curves. Figure 12a shows a comparison of the value of the loss curves of the cow feeding behaviour training data set taken from the front, and Figure 12b shows a comparison of the value of the loss curves of the cow feeding behaviour training data set taken from the top. As can be seen from the graphs, after 100,000 training cycles, the DRN-YOLO model achieves a perfect fit for both the front and top cow feeding behaviour training data sets, with no peak fluctuations and stable performance during the training process, while the YOLOv4 model experiences large fluctuations for both the front and top cow feeding behaviour training data sets, with a loss value break at around 80,000. The YOLOv4 model shows large fluctuations in both the front and top training datasets, with a break in the loss value at around 80,000 times and a peak fluctuation at around 55,000 times in the top training dataset, with a loss value of 4.8 or more. During the DRN-YOLO training process, the range of variation of loss values was small, generally controlled within 0.1, while the range of variation of loss values during the YOLOv4 training process was large, with the variation of loss values before and after of basically around 0.5–0.6. From the analysis of the loss curves, it can be seen that DRN-YOLO has a greater improvement compared to YOLOv4.

**Figure 13:**
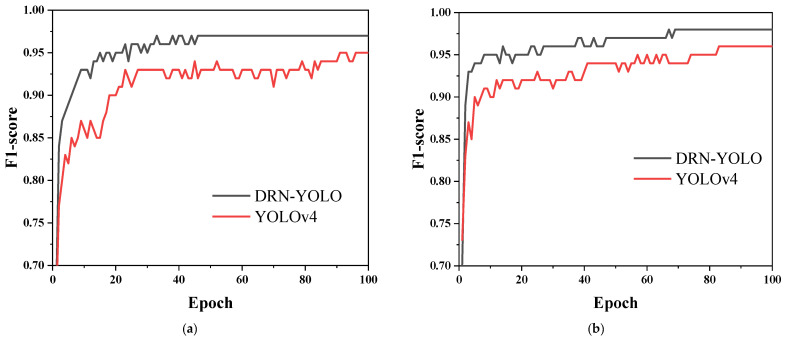
F1-score curves. (**a**) F1-score training data from front; (**b**) F1-score training data from top.

F1-score is one of the important indicators to evaluate the goodness of the model. F1-score integrates the *accuracy* and *recall* rate of the model training, so it is very important to analyse the model F1-score. As shown in Figure 13a for the F1-score of the cow feeding behaviour dataset taken in front and Figure 13b for the F1-score of the cow feeding behaviour dataset taken from the top, the red curve indicates the F1-score of the DRN-YOLO model, and the orange curve indicates the F1-score of the YOLOv4 model. It can be seen that the F1-score of the DRN-YOLO model is 0.2–0.3 higher than the F1-score of the YOLOv4 model for 113 iterations, which also proves that the DRN-YOLO model performs better than the YOLOv4 model.

**Figure 14:**
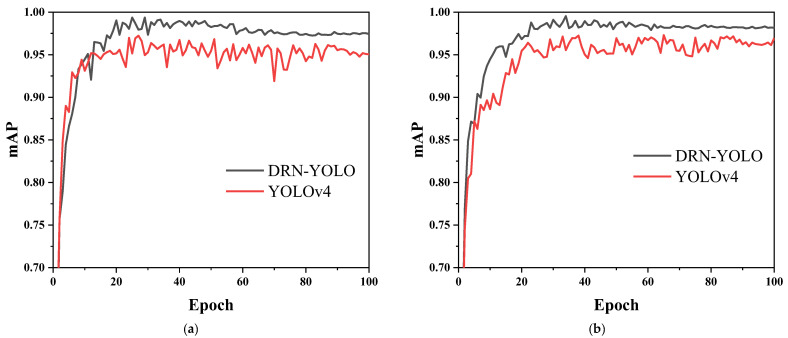
mAP curves. (**a**) *mAP* curve of training data from front; (**b**) *mAP* curve of training data from top.

The *mAP* value is an important indicator of the accuracy of the model in identifying object species. For example, Figure 14a shows the *mAP* values of the cow feeding behaviour dataset taken in front, and Figure 14b shows the *mAP* values of the cow feeding behaviour dataset taken from the top. The red curve indicates the *mAP* values of the DRN-YOLO model, and the orange curve indicates the *mAP* values of the YOLOv4 model. The *mAP* values of the DRN-YOLO model basically remained above 0.98, while those of the YOLOv4 model stayed between 0.95 and 0.96, demonstrating that the DRN-YOLO model is more accurate in identifying object species than the YOLOv4 model.

### 5.4. Comparison of DRN-YOLO with Classical Object Detection Algorithms

The performance of deep learning models needs to be compared between models [28]. To validate the effectiveness of cow feeding behaviour detection in the application of deep learning algorithms and to further analyse the performance of the DRN-YOLO model, we used three classical models, YOLOv4, SSD, and Faster RCNN, with *precision, recall, mAP,* and F1-score as evaluation metrics, for a comprehensive comparison with the DRN-YOLO model. A uniform dataset and test set were used for training and testing, with a uniform image input size of 416 × 416, while the experimental parameters were kept consistent. The results of the comparison tests for each model for the dataset of cow feeding behaviour taken in front and above are shown in Table 5 and Table 6.

The experimental results showed that the overall performance of the DRN-YOLO model was about 2% better compared to both the YOLOv4 model and the SSD model in the testing of the cow feeding behaviour datasets, whether taken in front or above, and was similar to the performance of the Faster RCNN model, which was only about 0.05% better; however, the Faster RCNN model is a two-stage detection model and the DRN- YOLO is a single-stage detection model. The two-stage model needs to go through both the RPN branch and the classification branch in the detection process, which causes the Faster RCNN model to use a longer time for detection than the DRN-YOLO model. In training and testing, the DRN-YOLO model detected the feeding behaviour of cows photographed in front with a *precision, recall, mAP,* and F1-score of 97.16%, 96.51%, 96.91%, and 96.83%, respectively, showing an improvement of 1.70%, 1.82%, 0.97%, and 1.76%, respectively, compared to YOLOv4. The DRN-YOLO model detected the feeding behaviour of cows filmed from above with 96.84%, 96.25%, 96.49%, and 96.55% *precision, recall, mAP,* and F1-score, respectively, showing an increase of 1.67%, 1.27%, 1.48%, and 1.48% compared to YOLOv4. It can be seen that DRN-YOLO has improved cow feeding behaviour detection compared to YOLOv4, the model detection performance is slightly higher than that of the two-stage detection model Faster RCNN, and the detection takes less time. The DRN-YOLO model was shown to be more comprehensive, faster, and more accurate in detecting cow feeding behaviour, which meets the requirement of accurate and fast cow feeding behaviour detection.

The recent studies on dairy cow feeding behaviour include Refs. [5,11]. Ref. [5] used CNN 2 to detect dairy cow feeding behaviour with a *precision* of 94.18% at 5800 iterations; Ref. [11] detected dairy cow feeding behaviour by sound and deep learning algorithm, and the final detection *precision, recall,* and F1-score were 79.3%, 79.7%, and 79.5%. Comparing DRN-YOLO with these two algorithms to analyse the detection performance of the DRN-YOLO algorithm shows that DRN-YOLO has a 2.98% improvement in *precision* compared to the detection method of [5], and 17.86%, 16.81%, and 17.33% improvement in *precision, recall,* and F1-score compared to the detection method of [11]. As can be seen, DRN-YOLO shows a large improvement compared with both dairy cow feeding behaviour detection algorithms, which validates the feasibility of DRN-YOLO for detecting dairy cow feeding behaviour.

### 5.5. Limitations Analysis

The limitation of the model algorithm proposed in this paper is that only the ablation test is carried out for each detection module, and it is not compared with other target detection and target tracking models, which makes the model lack comparison data. In addition, in the process of identifying the feeding behaviour of dairy cows, this model is quick to misjudge the grass arching behaviour as a feeding behaviour. After preliminary analysis, this is because the characteristics of cow grass arching behaviour and feeding behaviour are highly similar, and grass arching behaviour is generally completed in a very short time, the model tends to judge grass arching behaviour and feeding behaviour as the same behaviour. Therefore, the model has not effectively excluded the redundant state of the arch grass behaviour.

In future research, cow feeding behaviour can be further subdivided to identify and detect indistinguishable behaviours such as chewing, swallowing, regurgitation, and pushing using deep learning techniques; the feed depth information collected by the ZED binocular camera can be used to analyse the feed consumption of cows within a certain period of time, thus realising real-time calculation of cow feeding, and when combined with the research results of this paper, the design and development of an automatic monitoring system for dairy cows’ feeding behaviour can be realised to achieve the requirement of long-term monitoring of dairy cows’ feeding behaviour.

## 6. Conclusions

The recognition method of cow feeding behaviour based on DRN-YOLO algorithm and edge computing technology was proposed in this study, ultimately achieving accurate and fast detection of cow feeding behaviour in farm feeding environments. The results of the experiment are as follows.

(1) This study tested the performance enhancement of the YOLOv4 model by different modules to verify the effectiveness of the improved model. For example, using the DRNet backbone network alone in a single module improved the model the most, with 1.03%, 0.78%, and 0.84% improvement in *precision, recall,* and *mAP,* respectively, and DRN-YOLO improved 1.70%, 1.82%, and 0.97% in *precision*, *recall,* and *mAP,* respectively, compared to the YOLOv4 model. The DRN-YOLO algorithm was compared with the YOLOv4 model, SSD model, and Faster RCNN model in terms of computational efficiency, and the algorithm studied in this paper showed faster detection compared to the two-stage Faster RCNN model.

(2) Deep learning–based recognition of cow foraging behaviour was achieved by relying on edge computing technology, but there are still some limitations. Future research can further subdivide cow foraging behaviour to identify and detect indistinguishable behaviours such as chewing, swallowing, regurgitating, and arching using deep learning techniques.

## Figures and Tables

**Table 1:** Parameters of NVIDIA JetsonTX2.

Items	Parameters
GPU	NVIDIA Pascal^TM^ architecture with 256 CUDA cores
CPU	Dual-core Denver2 64-bit CPU and quad-core ARM A57 Complex
Video encoding/decoding	4 K × 2 K 60 Hz encoding (HEVC); 4 K × 2 K 60 Hz decoding (12-bit support)
Video memory	8 GB 128-bit LPDDR4 59.7 GB/s
Display	2 DSI ports, 2 DP 1.2/HDMI 2.0 ports/eDP 1.4 ports
CSI	CSI support for up to 6 cameras (2 channels) CSI2 D-PHY 1.2 (2.5 Gbps per channel)
PCIE	Gen 2|1 × 4 + 1 × 1 or 2 × 1 + 1 × 2
Data storage	32 GB eMMC, SDIO, SATA
Other	CAN, UART, SPI, I2C, I2S, GPIO
Connectable	1 Gigabit Ethernet, 802.11ac WLAN, Bluetooth
Mechanical	50 mm × 87 mm (400-pin compatible board-to-board connector)

**Table 2:** Number of datasets labelled with cow feeding behaviour.

Shooting Direction	Number of Training Datasets	Number of Test Datasets	Number of Feeding Behaviours	Number of Chewing Behaviours	Number of Pushing Behaviours
Front	4484	758	5684	792	1613
Top	4320	726	6958	960	1946

Note: The number of training sets and the number of test sets were the number of pictures for training and testing, and the number of feeding behaviours, chewing behaviours, and pushing behaviours were the number of labelled boxes for feeding behaviour, chewing behaviour and pushing behaviour in the training and test sets.

**Table 3:** The results of training on the dataset from the front-shot dairy cows’ feeding behaviour.

YOLOv4	DRNet	Four-Feature Scale	SPP	mAP (%)	Precision (%)	Recall (%)	F1-Score
√				95.13	95.46	94.69	95.07
√		√		95.86	96.03	95.24	95.63
√		√	√	96.27	96.42	95.75	96.08
√	√			96.16	96.24	95.53	95.88
√	√	√		96.58	96.72	96.17	96.44
√	√	√	√	96.91	97.16	96.51	96.83

**Table 4:** The results of training on the dataset from the above-shot dairy cows’ feeding behaviour.

YOLOv4	DRNet	Four-Feature Scale	SPP	mAP (%)	Precision (%)	Recall (%)	F1-Score
√				95.01	95.17	94.98	95.07
√		√		95.53	95.73	95.03	95.38
√		√	√	95.97	96.12	95.48	95.80
√	√			95.69	95.96	95.26	95.61
√	√	√		96.08	96.44	95.70	96.07
√	√	√	√	96.49	96.84	96.25	96.54

**Table 5:** The results of testing on the dataset from the front-shot dairy cows’ feeding behaviour.

Model	Precision (%)	Recall (%)	*mAP* (%)	F1-Score (%)	Time (ms)
YOLOv4	95.46	94.69	95.13	95.07	31.17
SSD	95.34	95.08	95.14	95.21	-
Faster RCNN	97.11	96.50	96.88	96.80	160
DRN-YOLO(OURS)	97.16	96.51	96.91	96.83	22.65

**Table 6:** The results of testing on the dataset from the above-shot dairy cows’ feeding behaviour.

Model	Precision (%)	Recall (%)	*mAP* (%)	F1-Score (%)	Time (ms)
YOLOv4	95.17	94.98	95.01	95.07	31.17
SSD	95.14	94.96	95.04	95.04	-
Faster RCNN	96.81	96.21	96.43	96.51	160
DRN-YOLO(OURS)	96.84	96.25	96.49	96.55	22.65

## Data Availability

The raw data needed to reproduce these findings cannot be shared at this time, as these data are also part of further research.

## References

[1] Bareille N., Beaudeau F., Billon S., Robert A., Faverdin P. (2003). Effects of health disorders on feed intake and milk production in dairy cows. Livest. Prod. Sci..

[2] Fogsgaard K.K., Rontved C.M., Sorensen P., Herskin M.S. (2012). Sickness behavior in dairy cows during Escherichia coli mastitis. J. Dairy Sci..

[3] Fogsgaard K.K., Bennedsgaard T.W., Herskin M.S. (2015). Behavioral changes in freestall-housed dairy cows with naturally occurring clinical mastitis. J. Dairy Sci..

[4] Thorup V.M., Nielsen B.L., Robert P.E., Giger-Reverdin S., Konka J., Michie C., Friggens N.C. (2016). Lameness Affects Cow Feeding But Not Rumination Behavior as Characterized from Sensor Data. Front. Vet. Sci..

[5] Achour B., Belkadi M., Filali I., Laghrouche M., Lahdir M. (2020). Image analysis for individual identification and feeding behaviour monitoring of dairy cows based on Convolutional Neural Networks (CNN). Biosyst. Eng..

[6] Chen C., Zhu W., Norton T. (2021). Behaviour recognition of pigs and cattle: Journey from computer vision to deep learning. Comput. Electron. Agric..

[7] Shane D.D., White B.J., Larson R.L., Amrine D.E., Kramer J.L. (2016). Probabilities of cattle participating in eating and drinking behavior when located at feeding and watering locations by a real time location system. Comput. Electron. Agric..

[8] Pastell M., Frondelius L. (2018). A hidden Markov model to estimate the time dairy cows spend in feeder based on indoor positioning data. Comput. Electron. Agric..

[9] Porto S.M.C., Arcidiacono C., Giummarra A., Anguzza U., Cascone G. (2014). Localisation and identification performances of a real-time location system based on ultra wide band technology for monitoring and tracking dairy cow behaviour in a semi-open free-stall barn. Comput. Electron. Agric..

[10] Li W., Ji Z., Wang L., Sun C., Yang X. (2017). Automatic individual identification of Holstein dairy cows using tailhead images. Comput. Electron. Agric..

[11] Li G., Xiong Y., Du Q., Shi Z., Gates R.S. (2021). Classifying Ingestive Behavior of Dairy Cows via Automatic Sound Recognition. Sensors.

[12] Shen W., Sun Y., Zhang Y., Fu X., Hou H., Kou S., Zhang Y. (2021). Automatic recognition method of cow ruminating behaviour based on edge computing. Comput. Electron. Agric..

[13] Kang X., Zhang X.D., Liu G. (2021). A Review: Development of Computer Vision-Based Lameness Detection for Dairy Cows and Discussion of the Practical Applications. Sensors.

[14] Tian F., Wang J., Xiong B., Jiang L., Song Z., Li F. (2021). Real-Time Behavioral Recognition in Dairy Cows Based on Geomagnetism and Acceleration Information. IEEE Access.

[15] Campos D.P., Abatti P.J., Bertotti F.L., Hill J.A.G., da Silveira A.L.F. (2018). Surface electromyography segmentation and feature extraction for ingestive behavior recognition in ruminants. Comput. Electron. Agric..

[16] Liu T., Pang B., Ai S., Sun X. (2020). Study on Visual Detection Algorithm of Sea Surface Targets Based on Improved YOLOv3. Sensors.

[17] Jiang A., Noguchi R., Ahamed T. (2022). Tree Trunk Recognition in Orchard Autonomous Operations under Different Light Conditions Using a Thermal Camera and Faster R-CNN. Sensors.

[18] Porto S.M.C., Arcidiacono C., Anguzza U., Cascone G. (2015). The automatic detection of dairy cow feeding and standing behaviours in free-stall barns by a computer vision-based system. Biosyst. Eng..

[19] Bezen R., Edan Y., Halachmi I. (2020). Computer vision system for measuring individual cow feed intake using RGB-D camera and deep learning algorithms. Comput. Electron. Agric..

[20] Yang A., Huang H., Zheng B., Li S., Gan H., Chen C., Yang X., Xue Y. (2020). An automatic recognition framework for sow daily behaviours based on motion and image analyses. Biosyst. Eng..

[21] Lao F., Brown-Brandl T., Stinn J.P., Liu K., Teng G., Xin H. (2016). Automatic recognition of lactating sow behaviors through depth image processing. Comput. Electron. Agric..

[22] Shelley A.N., Lau D.L., Stone A.E., Bewley J.M. (2016). Short communication: Measuring feed volume and weight by machine vision. J. Dairy Sci..

[23] Singh D., Kumar V., Kaur M. (2021). Densely connected convolutional networks-based COVID-19 screening model. Appl. Intell..

[24] He K., Zhang X., Ren S., Sun J. Deep Residual Learning for Image Recognition. Proceedings of the 2016 IEEE Conference on Computer Vision and Pattern Recognition (CVPR 2016).

[25] Huang Z., Wang J., Fu X., Yu T., Guo Y., Wang R. (2020). DC-SPP-YOLO: Dense connection and spatial pyramid pooling based YOLO for object detection. Inf. Sci..

[26] Liu S., Qi L., Qin H., Shi J., Jia J. Path Aggregation Network for Instance Segmentation. Proceedings of the 2018 IEEE Conference on Computer Vision and Pattern Recognition (CVPR 2018).

[27] Lin T.Y., Dollár P., Girshick R., He K., Hariharan B., Belongie S. Feature Pyramid Networks for Object Detection. Proceedings of the 2017 IEEE Conference on Computer Vision and Pattern Recognition (CVPR 2017).

[28] Zhang H., Li F., Liu S., Zhang L., Su H., Zhu J., Shum H.Y. (2022). DINO: DETR with Improved DeNoising Anchor Boxes for End-to-End Object Detection. arXiv.

